# The Causes of Insanity*Read before the Miami Valley Medical Society, November 1, 1898.

**Published:** 1898-12

**Authors:** Philip Zenner

**Affiliations:** Cincinnati; Lecturer on Diseases of the Nervous System in the Medical College of Ohio


### THE CAUSES OF INSANITY.*

BY PHILIP ZENNER, A.M., M.D., CINCINNATI.

Lecturer on Diseases of the Nervous System in the Medical College of Ohio.

GENTLEMEN: The subject upon which I have been asked to read to-day is, indeed, an old one, but yet so important that it may not be without profit to present it for your consideration.

Insanity is supposed to be, essentially, a disease of civilization. To what extent it has occurred, and occurs, among primitive peoples can not be determined with any accuracy; certainly far less than with us. Statistics seem to prove its rapid increase in our own day. But such observations are largely delusive, for they are based chiefly on the number of inmates in public and private asylums, and this increase in number is due to the multiplication of asylums, the greater readiness to resort to the same—which brings many insane out of hiding—and the prolongation of life through better treatment, which increases the number of old people, and thereby the sum total of the insane.

It is still possible, as is generally believed, that there is an actual increase of insanity, for some great causes seem to be acquiring a greater influence; for instance, cities grow more rapidly, factory life becomes more prevalent, the struggle for life is getting fiercer, syphilis more widespread, etc. There is no doubt that some mental disorders, notably paresis, have increased in recent years.

At the same time there is a growth of other influences that tend to lessen disease: improved hygiene, better food, clothing, dwellings, etc.; also greater security, as well as the feeling of security as regards epidemics, war, famine, and the like.

Insanity is found at all periods of life. To a certain extent the etiology is characteristic of the period. The mental disease of childhood is rather that of mental arrest, varying from slight imbecility to profound idiocy. Its basis is usually structural disease from inherited or other congenital conditions, or the accidents or diseases of birth and infancy.

The insanity of puberty and the climacterium occurs mostly in predisposed subjects, that predisposition being most commonly an hereditary taint.

The mental disorders of old age, like those of childhood, have usually a structural basis; in this instance disease of blood-vessels, or changes in the brain. In adult life, the age most prone to mental disease, the latter is chiefly due to extrinsic conditions, alcohol, syphilis, and the excesses and trials of life.

Both sexes are about equally attacked, though there is some

*Read before the Miami Valley Medical Society, November 1, 1898.

difference as to the most effective causes in each. The changes of puberty and the climacterium are more frequently disastrous to the female than the male. The former has also to contend with pregnancy, parturition, and lactation, as well as, ofttimes, a life with little aim and purpose. In the male we find more commonly alcohol, tobacco, syphilis, sexual excesses and abuses, while he must usually bear the brunt of the struggle for existence.

HEREDITY.

When we come to special causes, we must place at the head of the list heredity. It is so difficult to elicit facts as regards hereditary taint, on account of the desire to conceal them, or ignorance on the part of the family or patient, that there have been great discrepancies in statistics on the subject. The proportion of the insane who have an hereditary taint has been estimated all the way from 10 to 90 per cent. We will probably not go far amiss if we put it between 30 and 40 per cent.

The disease may appear in the same form in parent and offspring; in fact, the resemblance may be so great that even the same ideas and delusions reappear. A striking instance of this order is the frequent occurrence of suicide in a family, the separate acts often at a distance in time and space, and apparently independent of each other. More frequently the disease is not alike in different generations. For instance, epilepsy in the parent is followed by brain disease in another form in the offspring. In the same way different forms of insanity, alcoholism, epilepsy, and other nervous diseases, tuberculosis, etc., become related to one another.

Morel formulated a law of progressive degeneracy, according to which light nervous or mental troubles in the first generation are followed by gradually more profound mental disease in the second and third generations, and, finally, by idiocy and sterility in the fourth. But such a typical history is rarely found. Usually there are changes for better or worse from one generation to another, according as an admixture of healthy blood or other favorable conditions come in on the one hand, or alcohol, crime, or other unfavorable conditions come in on the other.

The manner of transmission of disease varies also. The hereditary taint may consist of actual disease, or merely a predisposition to it. In the first instance structural changes may or may not be detected. But there is, at least, some pathological state of the nervous system, manifested from infancy by peculiarities of character and the like. Such a condition is usually the foundation of certain forms of insanity, notably paranoia and moral insanity.

The predisposition to disease probably consists of an inherent weakness in the organism, a lessened power of resistance, which renders it a prey to disease where a normal nervous system would escape. Doubtless many individuals with such heredity pass through life unscarred, because they have never been subjected to specially injurious influences. Again, this class is often attacked by mental disease at puberty, the climacterium, in child ed, after infectious and other diseases, and succumbs the more easily in mature years to alcohol, syphilis, psychic disturbances, excesses, etc.

The likelihood of transmission of disease is greater if both, than if only one, parent is affected, and greater when the disease occurs in the parent than when only found in more distant ancestry. The likelihood of transmission depends also on how deeply rooted is the disease in the parent's system. An acute attack of mania or melancholia in an otherwise normal being may have no effect on the offspring, whereas, much more trivial manifestations, as nervousness and peculiar traits of character, which are, as it were, a part of the individual's nature, will very likely leave their imprint.

Epilepsy in childhood is likely to stamp the nature of the individual and mark the offspring, while the same disease, occurring late in life, may leave the offspring unscathed. The more frequently a disease has occurred in the ancestry, the more deeply rooted it is likely to be in the individual, and, therefore, the more likely its farther transmission.

Such facts should be borne in mind by the physician when he is consulted on the subject of marriage. The much mooted question, the effect of the marriage of cousins, is influenced rather by whether or not there be an hereditary taint, and, if so, its degree on each side, than the mere fact of consanguinity.

In connection with transmission of disease, I will mention the bad effects of alcoholism, or other abnormal states at the time of procreation, and of disease of the mother during pregnancy.

### OTHER PREDISPOSING CAUSES.

In addition to heredity, often in conjunction with it, we find many other predisposing causes of disease. Convulsions, and intra-cranial inflammation in infantile life, trauma in childhood, as well as before and during birth, other diseases of intra-uterine and infantile life, though at the time they may have terminated in apparent recovery, are likely to have left their stamp on the individual, a predisposition to future disease. The predisposition may also be acquired through the manner of life, the training of the child, etc. Our systems of education are, doubtless, responsible for not a small part of the ills of later life. It is especially unfortunate that children of weakly nature and with hereditary taints should be subjected to the same school discipline as those with vigorous constitutions.

Perhaps we should look upon an attack of mental disease as creating a predisposition to the same. At least recurrences are common. I have quite a number of individuals under observation who have had repeated attacks. In some the interval of health was as much as ten years. In all, the period of health was too long to look upon their cases as a continuous disease with intervals of apparent health as occurs in circular insanity.

PSYCHIC CAUSES.

Among the most common exciting causes of insanity are psychic disturbances. These are usually painful and depressing emotions. Joyful emotions rarely do harm, nor is overwork in itself a cause of mental disease. It is only when accompanied by continual worry, or the like, that it finally causes a mental breakdown, perhaps insanity. One of the most injurious causes of this kind is grief, most commonly that for the loss of husband, wife, or child. Almost equally disastrous is disappointment, especially if it follows a long period of emotional strain, and is attended by a sense of mortification or humiliation. Such causes are especially effective if cherished in secret, for tears and wailing are an external discharge, which do much to lessen internal danger.

It is usually the long continuance of mental pain, the ceaseless grief, the cankering care, which finally leads to mental disease. More rarely the great intensity of the emotional state, fright, terror, or the like, cause a rapid outburst of disease. In all instances mental causes are more disastrous where there is a predisposition to mental disease.

TRAUMA.

Among the causes of insanity trauma takes an important place. As already stated, trauma during uterine life, in the act of delivery, and in infancy, produces pathological states of the nervous system, which predispose to insanity in later years. How often it produces insanity in mature years is not very clear. Doubtless it is often assigned as a cause without reason, and, again, may have escaped recognition altogether when pronounced symptoms first appeared long after the trauma occurred.

The immediate cause of the disease is usually a structural lesion, as fracture of bone, injury and inflammation of membranes, hemorrhages, etc. Of most consequence is the disturbed circulation of the cortex and impairment of its nutrition. Often mental disease follows comparatively slight injury of the head, or is a part manifestation of what is spoken of as railway spine or traumatic neurosis. Here we must suppose shock, fright, general disturbance of health, and, perhaps, a predisposition to disease, to be the chief factors in its production. Mental disease following trauma may assume many forms. The most common picture, perhaps, is irritability, craving for alcohol, and dementia. Not rarely it appears as epileptic insanity.

### SYPHILIS.

Syphilis is becoming one of the great scourges of civilization. How great is its influence in the production of organic nervous disease was only recently recognized, when it was observed to be an important etiological factor in the production of locomotor ataxia and paresis. In cases of the latter disease a prior history of syphilis has been found in as many as 75 per cent. by some observers. Possibly we should consider syphilis as rather a predisposing than exciting cause. At least we do not find characteristic syphilitic lesions in paresis, nor is it benefited by anti-syphilitic treatment. Paresis and pseudo-paresis—a form of cerebral syphilis which very much resembles paresis—are the most common forms of insanity due to syphilis. In the early periods of syphilitic infection, mania, melancholia, etc., sometimes occur, but their relation to syphilis is not altogether clear. The ordinary forms of brain syphilis, gumma, etc., may present mental symptoms, but these cases are scarcely to be classed with insanity.

### INFECTIOUS DISEASES.

The acute infectious diseases are not rare causes of insanity. Chief among these are the acute exanthemata, influenza, and typhoid. I shall merely refer to the mental disease following typhoid; for the latter is more frequently attended by mental disorder than the other infections, and at the same time the mental trouble is of greater severity and of less favorable prognosis.

The occurrence of mental symptoms in typhoid is very common. For the larger part we have only febrile delirium, but in quite a number of cases, perhaps from 6 to 8 per cent., mental disease continues for a long period, and one-half of these remain permanently insane. According to some observers one per cent. of the inmates of insane asylums owe their mental disease to typhoid. The immediate cause of the mental disease is more difficult to determine. In most instances it appears during the height of the fever, less frequently it begins after the fever has subsided, and in rare instances it sets in before the fever. Elevated temperature, the typhoid germ, disturbances in the cerebral circulation, œdema of the brain, degeneration of nervous tissue, may all play a part in the production of mental symptoms. Heredity, or other predisposition, often favors the mental outbreak.

It would tax your patience too much to describe thus fully still other causes of insanity. I shall, therefore, do little more than mention them.

### OTHER DISEASES.

Other diseases than those mentioned cause insanity more or less frequently through febrile disturbances, anæmia, impaired nutrition, pain and suffering, as well as by their being the source of worry, anxiety, fear, and the like.

In rheumatism and gout changes have been found in the meninges and cerebral vessels, accounting in part for the mental disorder occasionally attending these diseases. In some instances mental and rheumatic symptoms have interchanged with each other. Insolation may be followed by insanity, usually of transient character. Tuberculosis, heart disease, Bright's disease, cause insanity in a comparatively small number of cases. That attending Bright's is usually due to uræmia. Various functional and organic nervous diseases—neuralgia, cholera, locomotor ataxia, brain disease—may be attended by insanity. Epilepsy very often produces mental disorder. Periodical attacks of insanity, frequent changes in character, irritability, or more profound disturbances, and finally dementia, belong to the common history of this disease. Exopthalmic goiter is, also, very frequently complicated by mental disease. Myxœdema, in which the mental manifestations are a prominent part of the disease, is, fortunately, losing its sinister aspect under the new treatment with the thyroid. Diseases of the digestive organs are not unimportant factors in the production of mental disease, through unimpaired nutrition, disturbed circulation, and, possibly, the presence of toxic agents. The public attribute to disease of the sexual organs more influence in this direction than is their due. They probably play but a subordinate role in the production of insanity.

### TOXIC AGENTS.

It remains but to mention some toxic agents or drugs. First comes alcohol, the most fruitful source of mental disease. Though the cases are countless where this cause is manifest, it must not be forgotten that there are instances where the origin of the trouble is unknown. I have seen quite a number of cases of insanity in secret drinkers, in which the cause long eluded detection. The symptoms manifested may be quite varied. The other important agents of this character are opium, cocaine, chloral, ergot, the bromides, mercury, etc.

### EXCESSES.

Excesses in venery, onanie, abnormal sexual excitement, etc., probably produce disease through the exhaustion induced. They are to a greater extent predisposing than exciting causes. Often they are a manifestation of disease, instead of a cause. In those who have already a predisposition to disease, such excesses may become exciting causes.

In the examination of a patient, if we inquire carefully into his manner of life and all else, we will generally find that not a single cause, but a number, have conspired together to bring on the mental disease.—*Ohio Medical Journal.*

